# Prognostic value of CD44 expression in renal cell carcinoma: a systematic review and meta-analysis

**DOI:** 10.1038/srep13157

**Published:** 2015-08-19

**Authors:** Xintao Li, Xin Ma, Luyao Chen, Liangyou Gu, Yu Zhang, Fan Zhang, Yun Ouyang, Yu Gao, Qingbo Huang, Xu Zhang

**Affiliations:** 1 Department of Urology, State Key Laboratory of Kidney Disease, PLA Medical School, Chinese People’s Liberation Army General Hospital, Beijing, China

## Abstract

CD44 is a marker of cancer stem-like cells in renal cell carcinoma (RCC). However, the prognostic value of CD44 in RCC remains controversial. This study evaluated the correlation of CD44 expression with the clinicopathological features of RCC through a meta-analysis. We systematically searched PubMed, ISI Web of Science and Embase for relevant studies until February 2015. We collected and analysed data on clinical stage, Fuhrman grade, microvascular invasion, recurrence, five-year overall survival (OS), disease-specific survival (DSS) and disease-free survival (DFS). Twenty studies involving 1672 patients satisfied the inclusion criteria. Results showed that high CD44 expression in RCC was a poor prognostic marker for five-year OS (RR = 0.69, 95% CI 0.60–0.78) in a fixed-effects model and for five-year DSS (RR = 0.46, 95% CI 0.27–0.80) and five-year DFS (RR = 0.63, 95% CI 0.43–0.93) in a random-effects model. CD44 expression also correlated with Furhman grade (RR = 0.61, 95% CI 0.48–0.77), tumour recurrence (RR = 7.42, 95% CI 3.74–14.70) and MVI (Microvascular invasion) (RR = 3.63, 95% CI 1.97–6.71). This meta-analysis suggests that CD44 is a prognostic marker in RCC. High CD44 expression correlates with high Fuhrman grade, recurrence, MVI and poor prognosis.

Renal cell carcinoma (RCC) represents approximately 2%–3% of all human malignancies and is the second leading cause of death in urological malignant neoplasms^1^. Approximately 25%–30% of patients present with metastatic disease at the time of diagnosis, and metastatic RCC (mRCC) is a treatment-resistant malignancy^2^. Target therapy of metastatic RCC has dramatically advanced in the past few years, but most mRCC patients still eventually die of the disease^3^. To date, a few markers for RCC have been identified to distinguish patients with aggressive forms from those with good outcomes.

A recent study has reported that cancer stem cells (CSCs) cause cancer heterogeneity and regulate the initial, progression, metastasis and eventual recurrence of cancer^4^. CSCs possess self-renewal and high tumour-initiating potentials when inoculated in immunodeficient mice^5^. Thus, eliminating CSC is critical in cancer therapy^6^. Furthermore, CSC markers, such as CD44, CD133 and ALDH1A1, are potential indicators of RCC prognosis^7^,^8^,^9^. Among these CSC markers, CD44 is the most frequently reported in RCC. Several studies similarly utilised CD44 positivity to isolate cells with stem cell-like and cancer-initiating properties from other cancer cells^10^,^11^,^12^.

CD44 receptor is a transmembrane glycoprotein with different variant isoforms because of the alternative splicing of variable exons^13^. This receptor is located on chromosome 11p13 and consists of at least 20 exons^14^. Among the variant isoforms, the CD44 standard form (CD44s) is the main receptor for hyaluronate^15^. CD44s bound to hyaluronan reportedly affects various tumour biological activities, such as tumour progression, proliferation and metastasis^16^. Other isoforms are called CD44 variants (CD44v), which are generated by alternatively spliced transcripts^13^. To date, CD44s remains the most reported isoform in RCC. Other CD44 variants include CD44v3, CD44v5, CD44v6 and CD44v10. Many studies demonstrated that CD44 exerts a negative prognostic value in RCC, but some studies disclosed that CD44 elicits no effect on the survival of RCC patients^17^,^18^. Insufficient samples and some other factors resulted in controversial results. In the present study, we performed a meta-analysis to determine the prognostic value of CD44 in RCC. The correlation between CD44 expression and other clinical features was also analysed.

## Materials and Methods

### Search Strategy

A systematic literature search was performed in the electronic databases Pubmed, ISI Web of Science and Embase until February of 2015. Search terms included ‘CD44’, ‘renal or kidney’ and ‘tumour, cancer, neoplasm or carcinoma’. The titles and abstracts of potential references were carefully scanned to exclude irrelevant articles. The remaining articles were evaluated to identify research that contained the topic of interest, and full texts were reviewed in depth afterwards.

### Selection Criteria

The studies included in the present meta-analysis were randomised controlled studies or observational studies (case–control or cohort) that evaluated the association between the expression of CD44 and the prognosis or the clinical data of RCC. Studies were included if they (1) focused on the clinical features or prognosis of RCC; (2) detected CD44 protein expression by immunohistochemistry; and (3) analysed the correlation of CD44 expression with clinical features and survival outcomes [(disease-free survival (DFS), disease-specific survival (DSS) or overall survival (OS)]. Articles were excluded on the basis of the following criteria: (1) non-English papers; (2) review articles or case reports; (3) The study of CD44 mRNA expression by RT-PCR; (4) The study is focused on animal models or cancer cells; (5) The study didn’t analyze the CD44 protein expression and the clinical features and survival outcome; (6) the full text was unavailable.

All evaluations were independently performed by two reviewers to ensure the accurate inclusion of studies. When several studies containing overlapping data emerged, the one with the largest data set was adopted. If the original data were not provided in the text, we contacted the authors for the data necessary to conduct the meta-analysis.

### Data Extraction

All data were extracted by two independent reviewers. Disagreements in data extraction were resolved by reaching a consensus in accordance with the original article. The following relevant data were extracted in a predefined table: author, year, country, patient number, detection method, follow-up duration, antibody used, cut-off score used, tumour pathological type, staining patterns, high CD44 expression, tumour clinical stage, Fuhrman grade, tumour recurrence, microvascular invasion, five-year DFS, five-year DSS and five-year OS. Considering that some studies displayed survival data with a Kaplan–Meier curve, we used GetData Graph Digitizer 2.26 (http://getdata-graph-digitizer.com/) to digitise and extract survival data.

The cut-off score for CD44 positivity varied across different studies. Thus, we defined the CD44 positive group in accordance with the standards indicated in the original article. Several studies followed-up patients for either five-year DFS or five-year DSS. In this meta-analysis, we analysed both five-year DFS and five-year DSS.

### Statistical Analysis

Stata version 12.0 (StataCorp LP, TX) was used in this meta-analysis. The statistical analysis was performed according to the guidelines proposed by the Meta-analysis of Observational Studies in Epidemiology group. ORs with 95% CI were used to evaluate the correlation between CD44 expression and clinicopathological features, including tumor clinical stage, Fuhrman grade, tumor recurrence and microvascular invasion. RRs with 95% CI were used to evaluate correlation between CD44 expression and 5-year DFS, 5-year DSS and 5-year OS. The heterogeneity among studies was measured using the Q test and I2 test. A fixed-effects model was used in the absence of significant heterogeneity; a random-effect model was used otherwise. Begg’s and Egger’s tests were performed to identify the potential publication bias. P < 0.05 was considered to indicate statistical significance. All P values are two-tailed. Sub-group analyses were performed to investigate the association of CD44 expression with clinical features and prognosis in terms of geographic area, staining pattern, cutoff of staining, sample size and follow-up time. Considering that the CD44 variants CD44v3, CD44v5, CD44v6 and CD44v10 were only investigated in one or two studies, we only performed pooled analysis on CD44s. Furthermore, a sensitivity analysis was performed to examine the robustness of the pooled results. The meta-analysis followed the standard PRISMA checklist (Supplemental Table 1).

## Results

### Search Results

**Figure 1:**
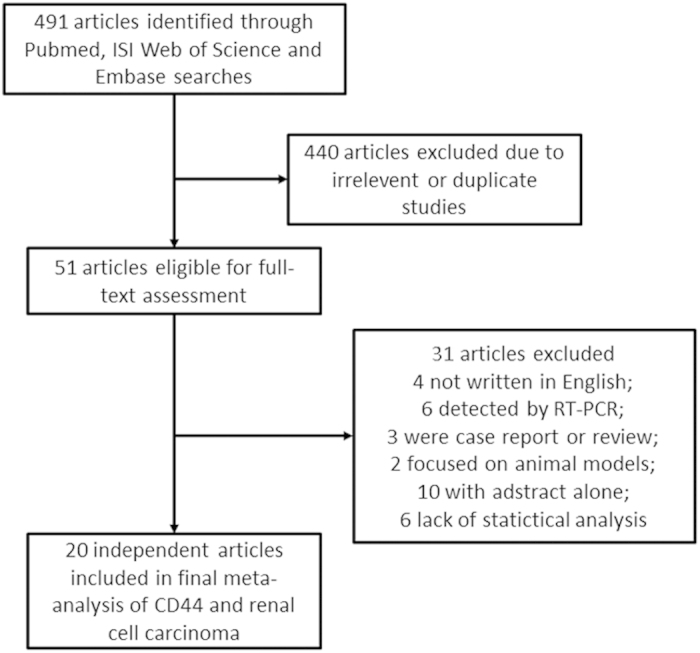
Flowchart of selection of studies for inclusion in meta-analysis.

A total of 491 articles were retrieved using the search strategy (Fig. 1). After the initial evaluation of the title and abstract, 440 articles were excluded because of their irrelevance and duplication. The remaining articles were viewed in full text. Among the 51 articles, 30 were excluded, including 4 not written in English, 6 detected by RT-PCR, 3 case reports or reviews, 2 focused on animal models, 10 with abstract alone and 6 without statistical analysis. Finally, 20 studies with 1672 patients were included in the meta-analysis. All of the included studies evaluated CD44 expression relevant to the clinical feature or prognosis of RCC through immunohistochemical staining.

### Characteristics of Eligible Studies

All features of the 20 studies are listed in Supplemental Table 2. Among the studies, three originated from France, three from China, two from the United States, two from Japan, two from Croatia, two from Korea, one from Spain, one from Germany, one from Greece, one from Turkey, one from Iran and one from Brazil. A total of 1672 patients with a median of 74 patients (from 38 to 173) per study were included. The TNM stage was reported in 8 studies, and the Furhman tumour grade was reported in 13 studies. The recurrence and microvascular invasion rates were both reported in two studies. Patient outcome was reported in 17 studies (five-year DSS in 5 studies, five-year DFS in 6 studies and five-year OS in 10 studies).

All studies applied immunohistochemical staining to detect CD44 expression. Eighteen studies used the anti-CD44s or anti-CD44 antibody. The median percentage of patients with CD44-positive expression was 36.5% (16.4%–87.5%). Five studies used the anti-CD44v6 antibody. The median percentage with CD44v6 positive expression was 16.2% (3%–40.0%). Two studies used the anti-CD44v5 antibody with positive expression rates of 92.5% and 6.4%. Two other studies used the anti-CD44v3 and anti-CD44v10 antibodies with positive expression rates of 6.3% and 36.7%, respectively. This pooled analysis focused on studies that used the anti-CD44s or anti-CD44 antibody because the studies that used other variant isoforms were limited to correlation analysis. All patients received radical or partial nephrectomy. None of the patients received radiotherapy or chemotherapy before or after surgery.

### Correlation of CD44 with Clinicopathological Features

Correlation results between CD44 and clinical features are presented in Table 1. Overall analysis showed that high CD44 expression correlates with high Furhman grade (RR = 0.61, 95% CI 0.48–0.77), frequent post-operative recurrence (RR = 7.42, 95% CI 3.75–14.70) and MVI (RR = 3.63, 95% CI 1.97–6.71) (Supplemental Figs 1–4).

Sub-group analyses were stratified on the basis of geographic area, staining pattern, cutoff of staining, sample size and follow-up time. Studies in both Asia and non-Asian countries indicated that CD44 expression correlates with Furhman grade but not with clinical stage. Studies with membrane staining alone demonstrated that CD44 expression is associated with clinical stage (RR = 0.48, 95% CI 0.29–0.82) but not with Furhman grade (RR = 0.52, 95% CI 0.23–1.15). Meanwhile, studies with both membrane and cytoplasm staining suggested that CD44 expression correlates with Furhman grade (RR = 0.63, 95% CI 0.49–0.82) but not with clinical stage (RR = 0.86, 95% CI 0.61–1.22). In the cutoff of staining sub-group analysis, only studies with cutoff of over 20% showed that CD44 is related to high clinical stage (RR = 0.71, 95% CI 0.53–0.93) and Furhman grade (RR = 0.59, 95% CI 0.50–0.70). Similarly, only studies with a large sample size (over 58.5) indicated the correlation of CD44 with high clinical stage (RR = 0.69, 95% CI 0.53–0.88) and Furhman grade (RR = 0.59, 95% CI 0.44–0.77). Patients followed up with less than 68.5 months demonstrated a nearly significant correlation between CD44 and Furhman grade (RR = 0.68, 95% CI 0.43–1.07).

For other CD44 variant isoforms, CD44v6 was investigated in five studies^19^,^20^,^21^,^22^,^23^,^24^. However, four studies showed that CD44v6 positive rate is too low to perform an analysis. Only one study found high CD44v6 expression in 23 of 35 patients, and their results showed that CD44v6 expression correlates with nuclear grade, stage at diagnosis, post-operative metastasis and poor overall survival. Two studies focused on CD44v5 expression, and only one study concluded that the CD44v5 non-expression group has a higher mortality rate than the expression group^24^,^25^. One study with CD44v10 demonstrated that high CD44v10 expression correlates with high tumour grade, clinical stage and short survival^26^. The expression of CD44v3 was not detected in one study^22^.

### Impact of CD44 on Five-year OS, DFS and DSS Rates of RCC

**Figure 2:**
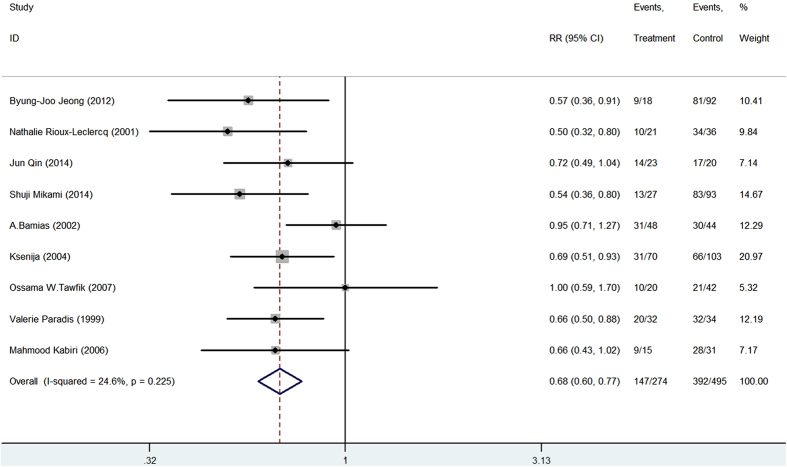
CD44 expression and 5-year OS in renal cell carcinoma patients.

The relationship between CD44 expression and RCC risk is illustrated in Fig. 2. The five-year OS rate was extracted from 9 studies. CD44 expression was statistically significantly associated with poor five-year OS rate in either the fixed-effects model (RR = 0.68, 95% CI 0.60–0.77) or the random-effects models (data not shown). No significant heterogeneity was observed among the studies (I^2^ = 24.6%, P = 0.23) (Supplemental Table 3). The five-year DSS and DFS rates were available in 4 and 6 studies, respectively. As shown in Supplemental Figs 5 and 6, high CD44 expression significantly correlated with poor five-year DSS rate (RR = 0.46, 95% CI 0.27–0.80, I^2^ = 83.7%) and five-year DFS rate (RR = 0.63, 95% CI 0.43–0.0.93, I^2^ = 86.3%) in the random-effects model.

Results of the sub-group analysis were consistent between Asian and non-Asian countries or different staining patterns with regard to five-year OS, DFS and DSS rates. High CD44 expression was significantly associated with poor five-year OS in every sub-group analysis but not with five-year DFS. The sub-group meta-analysis of studies with cutoff scores over 20% indicated that high CD44 expression was associated with poor five-year DFS rate (RR = 0.63, 95% CI 0.40–0.98, I^2^ = 84.1). High CD44 expression correlated with poor five-year OS in sub-groups with cutoff scores below (RR = 0.73, 95% CI 0.62–0.86, I^2^ = 44.9) or over 20% (RR = 0.63, 95% CI 0.52–0.77, I^2^ = 0). When grouped on the basis of sample size, both sub-groups supported that CD44 expression was a predictor for poor five-year OS and DSS. Sub-group meta-analysis of studies with follow-up periods of over 68.5 months showed that high CD44 expression was a predictor for both poor five-year OS and DSS. In studies with follow-up periods of less than 68.5 months, CD44 expression was not associated with five-year OS or DSS.

### Publication Bias Analysis

**Figure 3:**
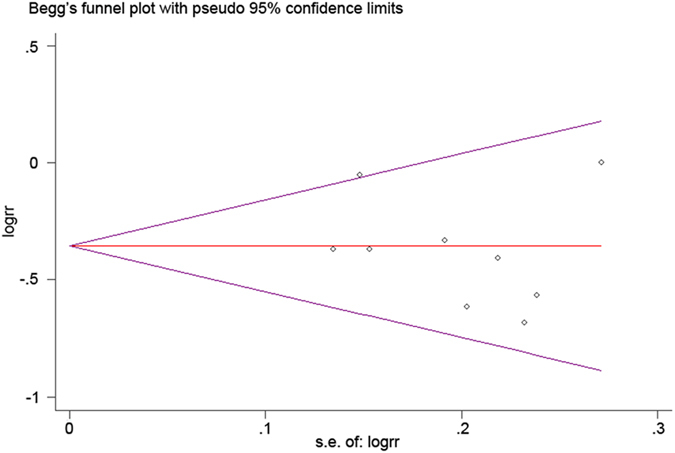
Begg’s test results of renal cell carcinoma patients’ 5-year OS rate.

**Figure 4:**
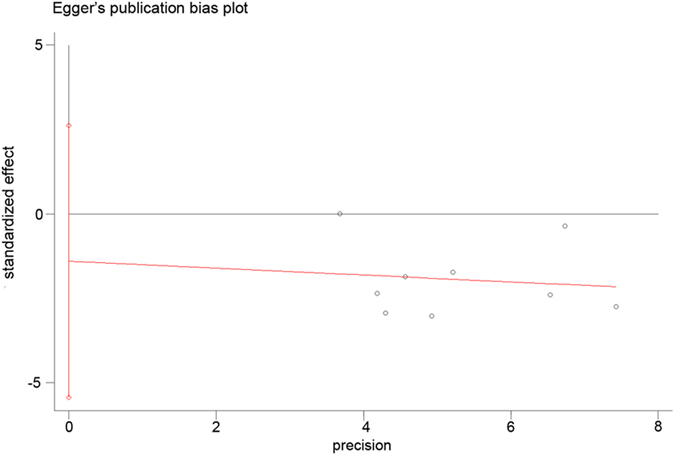
Egg’s test results of renal cell carcinoma patients’ 5-year OS rate.

We performed an analysis to evaluate potential publication bias for five-year OS rate. The funnel plots presented were almost symmetric, and no significant publication bias was detected (Begg’s P value = 0.602, Egg’s P value = 0.457) (Figs 3 and 4). The funnel plots showed that the selected studies had no apparent asymmetry.

### Sensitivity Analysis

**Figure 5:**
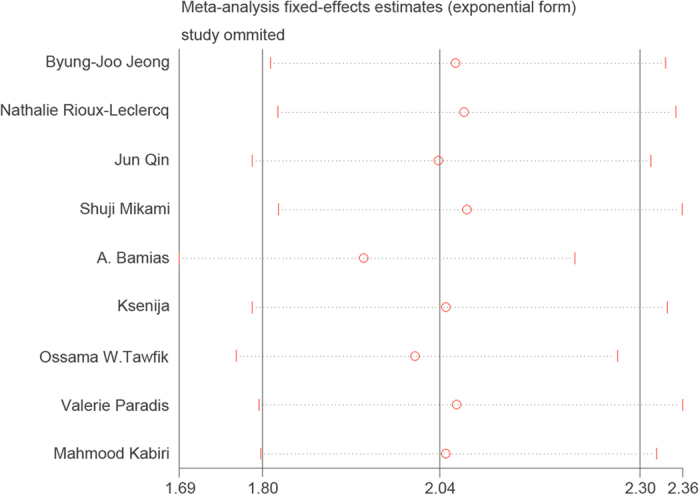
Sensitivity analysis of all the studies assessing 5-year OS rate.

A sensitivity analysis was performed in which one study was deleted at a time. Results are shown in Fig. 5. The pooled OR of five-year OS was not significantly changed, suggesting the robustness of the results.

## Discussion

According to CSC theory, CSCs possess self-renewal and high tumour-initiating potentials; thus, CSCs can drive cancer progression and metastasis^4^. CSCs were first identified in acute myeloid leukaemia to have differentiative and proliferative capabilities, as well as self-renewal potential^27^. To date, molecular markers remain lacking for RCC diagnosis and prognosis prediction. A significant fraction of RCC will still progress or metastasise after surgery. Therefore, searching for a useful biomarker for RCC remains urgent. CD44 is an important cancer stem cell marker in tumours and a poor prognostic marker in various cancers^28^,^29^,^30^,^31^,^32^,^33^,^34^. CD44 participates in the extravasation and migration of tumour cells. Pro-inflammatory cytokines stimulate CD44 expression and strengthen CD44–HA binding in endothelial cells, which can capture haematopoietic cells and tumour cells. The interactions of the CD44 cytoplasmic tail with the actin cytoskeleton can be activated through CD44–HA binding; thus, the migration of tumour cells is promoted^13^. In addition, CD44–HA binding can activate the ankyrin-based cytoskeleton and various Rho GTPase signalling pathways during tumour progression^35^. These studies indicate that CD44 can be a prognostic marker for RCC.

The present meta-analysis is the first study to systematically analyse the association between CD44 expression and RCC clinical features. Overall, CD44 expression significantly correlated with five-year OS, five-year DSS, five-year DFS, Furhman grade, tumour recurrence and MVI. Therefore, CD44 expression can be used as a marker to predict post-operative prognosis and tumour progression. Sub-group analysis showed that all of the analysis results were consistent across Asian and non-Asian RCC patients. When the cutoff of staining was over 20%, CD44 expression showed a prominent association with clinical stage, Furhman grade and five-year DFS. The above association was absent when the cutoff of staining was below 20%. These results further demonstrated that RCC with high CD44 was likely to become malignant. With regard to staining pattern, studies with CD44 membrane staining alone showed that its expression is significantly associated with clinical stage and five-year OS, but not with Furhman grade and five-year DFS. While both membrane and cytoplasm staining correlates with Furhman grade and five-year OS. Furthermore, the only study with cytoplasm staining pattern indicated no association between CD44 expression and five-year OS. Other studies about CD133 and CD166 expression in colorectal cancer come to the conclusion that the shift of CD44 from the cytoplasm to the membrane may transform renal cancer cells to an invasive phenotype^36^,^37^,^38^. But our results didn’t fully support the conclusion. When follow-up time exceeded 68.5 months, high CD44 expression significantly correlated with poor five-year OS and DSS. These findings suggest that the prognostic value of CD44 expression increases as the follow-up time is prolonged.

CD44 has different variant isoforms because of the alternative splicing of variable exons; CD44s is the most important CD44 isoform. However, studies for other CD44 variants in RCC are too limited to perform pooled analysis. Six studies evaluated the expression of CD44v6 on RCC, among which five studies found that the CD44v6 positive rate is too small to perform analysis. Only E de Alava *et al.* concluded that CD44v6 expression positively correlates with tumour progression and that its prognostic value is dependent on tumour stage at diagnosis^21^. Similarly, CD44v3 positive rate is also very low in one study^22^. Another study reported that CD44v10 expression significantly correlates with histological grade, clinical and pathological stages and cancer-specific survival^26^. Two studies obtained significantly different results on the expression rates (91.7% and 6.4%) of CD44v5^24^,^25^. Different from the other CD44 variants, low CD44v5 expression correlated with poor survival of RCC patients. These studies demonstrated the different functions of specific CD44 isoforms in cancer progression. Brown *et al.*^39^ demonstrated that the isoform switch to CD44s is essential for cells to undergo EMT and that the CD44s isoform can activate Akt signalling to drive EMT. The functions of the different variant isoforms of CD44 require further investigation.

The mRNA expression of CD44 is high in tumour tissues. Kan *et al.*^40^ initially found a higher mRNA expression level of CD44 in tumours than in normal kidneys. Hara *et al.*^41^ reported that CD44v8-10 is upregulated with the progression of non-papillary RCC. Andrew Chi *et al.*^42^ detected higher CD44 mRNA expression in metastatic RCC tissues than in non-metastatic tissues and that CD44s mRNA expression can be used as an independent prognostic indicator(OR = 1.02, 95%CI 1.01–1.05). These studies showed that CD44 mRNA expression is consistent with protein expression. But there are still limited studies focusing on association of CD44 mRNA expression and RCC prognosis and the available studies suggested that CD44s protein might be more important for RCC prognosis.

The evidence included in the present meta-analysis indicated CD44 expression as a poor prognostic marker in RCC. However, this study has several limitations. Firstly, the criteria to determine the positive or negative expression of CD44 varied across the included studies. So we performed subgroup analysis according to the cut-off criteria, which further proved that higher CD44 expression is associated with the advanced disease. Secondly, most of the studies included focused on CD44s expression, and the studies available for other CD44 variant isoforms were limited for analysis. Four out of five studies on CD44v6 demonstrated that its positive rate is too low to analyse the association between its expression and clinical features. Finally, only one study detected cytoplasmic CD44 expression in RCC and concluded that CD44 expression has no prognostic value in RCC. Therefore, the difference between CD44 membrane and cytoplasm staining in predicting RCC prognosis warrants further studies.

Despite the limitations listed above, the present analysis still revealed the prognostic value of CD44 expression in RCC. In specific, high CD44 expression correlated with poor five-year DFS, DSS and OS. CD44 expression also correlated with Furhman grade, tumour recurrence and MVI of RCC. Further studies assessing other CSC surface markers in combination with CD44 are required to evaluate their prognostic values in RCC.

## Additional Information

**How to cite this article**: Li, X. *et al.* Prognostic value of CD44 expression in renal cell carcinoma: a systematic review and meta-analysis. *Sci. Rep.* **5**, 13157; doi: 10.1038/srep13157 (2015).

## Supplementary Material

Supplementary Information

## Figures and Tables

**Table 1:** Results of meta-analysis on CD44 expression.

	Clinical Stage (I + II vs. III + IV)		Recurrence (positive vs. negative)		MVI (positive vs. negative)		Furhman Grade (grade I + II vs. grade III + IV)		5-Overall Survival (survive vs. death)		5-Disease Specific Survival (survive vs. death)		5-Disease Free Survival (survive vs. death)	
	N	RR(95%CI)	N	RR(95%CI)	N	RR(95%CI)	N	RR(95%CI)	N	RR(95%CI)	N	RR(95%CI)	N	RR(95%CI)
**Over all**	7	0.74(0.63, 1.04)	3	7.42(3.75, 14.70)	2	3.63(1.97, 6.71)	11	0.61(0.48, 0.77)	9	0.68(0.60, 0.77)	4	0.46(0.27, 0.80)	6	0.63(0.43, 0.93)
**Geographic area**														
1. Asian	2	0.63(0.32, 1.22)	NA	NA	NA	NA	4	0.47(0.31, 0.71)	4	0.60(0.49, 0.75)	1	0.58(0.39, 0.89)	3	0.55(0.22, 1.40)
2. Non-Asian	5	0.79(0.52, 1.21)	NA	NA	NA	NA	7	0.69(0.53, 0.90)	5	0.73(0.63, 0.86)	3	0.39(0.16, 0.92)	3	0.71(0.47, 1.08)
**Staining pattern**														
1. membrane	3	0.48(0.29, 0.82)	NA	NA	NA	NA	4	0.52(0.23, 1.15)	1	0.54(0.36, 0.80)	NA	NA	2	0.55(0.12, 2.42)
2. cytoplasm			NA	NA	NA	NA	NA	NA	1	1.00(0.59, 1.70)	NA	NA	NA	NA
3. membrane and cytoplasm	4	0.86(0.61, 1.22)	NA	NA	NA	NA	7	0.63(0.49, 0.82)	6	0.69(0.60,0.80)	NA	NA	3	0.76(0.49, 1.20)
**Cutoff of staining**														
1. < 20%	5	0.72(0.38, 1.37)	NA	NA	NA	NA	6	0.59(0.33, 1.06)	5	0.73(0.62, 0.86)	NA	NA	2	0.61(0.16, 2.42)
2. > = 20%	2	0.71(0.53, 0.93)	NA	NA	NA	NA	5	0.59(0.50, 0.70)	4	0.63(0.52, 0.77)	NA	NA	4	0.63(0.40, 0.98)
**Sample size**														
<58.5	3	0.73(0.22, 2.45)	NA	NA	NA	NA	4	0.68(0.39, 1.20)	5	0.68(0.57, 0.81)	2	0.27(0.17, 0.42)	NA	NA
≥58.5	4	0.69(0.53, 0.88)	NA	NA	NA	NA	7	0.59(0.44, 0.77)	4	0.69(0.58, 0.82)	2	0.69(0.56, 0.86)	NA	NA
**Follow time(month)**														
<68.5	4	0.63(0.47, 0.83)	NA	NA	NA	NA	5	0.68(0.43, 1.07)	3	0.88(0.70, 1.10)	2	0.33(0.04, 2.99)	2	0.93(0.66, 1.29)
≥68.5	2	0.68(0.54, 0.85)	NA	NA	NA	NA	5	0.53(0.41, 0.68)	5	0.64(0.54, 0.75)	2	0.45(0.27, 0.76)	3	0.53(0.20, 1.39)
