# Supplementary material for: Prognostic value of CD44 expression in renal cell carcinoma: a systematic review and meta-analysis
Source: Sci Rep. 2015 Aug 19;5:13157. doi: 10.1038/srep13157 (PMC4541415; doi:10.1038/srep13157)
Supplement: Supplementary Information [file srep13157-s1.pdf]

Prognostic value of CD44 expression in renal cell carcinoma: a systematic review and meta-analysis

Xintao Li[1#], Xin Ma[#], Luyao Chen, Liangyou Gu, Yu Zhang, Fan Zhang, Yun Ouyang, Yu Gao, Qingbo Huang, Xu Zhang*

Department of Urology, State Key Laboratory of Kidney Disease, PLA Medical School, Chinese People's Liberation Army General Hospital, Beijing, China.

# These authors contributed equally to this work.

* Corresponding author

Supplemental table 1 PRISMA checklist

Section/topic	#	Checklist item	Reported on page #
**TITLE**			
Title	1	Identify the report as a systematic review, meta-analysis, or both.	1
**ABSTRACT**			
Structured summary	2	Provide a structured summary including, as applicable: background; objectives; data sources; study eligibility criteria, participants, and interventions; study appraisal and synthesis methods; results; limitations; conclusions and implications of key findings; systematic review registration number.	2
**INTRODUCTION**			
Rationale	3	Describe the rationale for the review in the context of what is already known.	2,3
Objectives	4	Provide an explicit statement of questions being addressed with reference to participants, interventions, comparisons, outcomes, and study design (PICOS).	3,4
**METHODS**			
Protocol and registration	5	Indicate if a review protocol exists, if and where it can be accessed (e.g., Web address), and, if available, provide registration information including registration number.	
Eligibility criteria	6	Specify study characteristics (e.g., PICOS, length of follow-up) and report characteristics (e.g., years considered, language, publication status) used as criteria for eligibility, giving rationale.	4,5
Information sources	7	Describe all information sources (e.g., databases with dates of coverage, contact with study authors to identify additional studies) in the search and date last searched.	4
Search	8	Present full electronic search strategy for at least one database, including any limits used, such that it could be repeated.	4
Study selection	9	State the process for selecting studies (i.e., screening, eligibility, included in systematic review, and, if applicable, included in the meta-analysis).	4,5
Data collection process	10	Describe method of data extraction from reports (e.g., piloted forms, independently, in duplicate) and any processes for obtaining and confirming data from investigators.	5
Data items	11	List and define all variables for which data were sought (e.g., PICOS, funding sources) and any assumptions and simplifications made.	5
Risk of bias in individual studies	12	Describe methods used for assessing risk of bias of individual studies (including specification of whether this was done at the study or outcome level), and how this information is to be used in any data synthesis.	5,6
Summary measures	13	State the principal summary measures (e.g., risk ratio, difference in means).	5,6

Section/topic	#	Checklist item	Reported on page #
Synthesis of results	14	Describe the methods of handling data and combining results of studies, if done, including measures of consistency (e.g., I^2) for each meta-analysis.	5,6
Risk of bias across studies	15	Specify any assessment of risk of bias that may affect the cumulative evidence (e.g., publication bias, selective reporting within studies).	6
Additional analyses	16	Describe methods of additional analyses (e.g., sensitivity or subgroup analyses, meta-regression), if done, indicating which were pre-specified.	6
**RESULTS**			
Study selection	17	Give numbers of studies screened, assessed for eligibility, and included in the review, with reasons for exclusions at each stage, ideally with a flow diagram.	6
Study characteristics	18	For each study, present characteristics for which data were extracted (e.g., study size, PICOS, follow-up period) and provide the citations.	Page 7, Table S2
Risk of bias within studies	19	Present data on risk of bias of each study and, if available, any outcome-level assessment (see Item 12).	Table S3
Results of individual studies	20	For all outcomes considered (benefits or harms), present, for each study: (a) simple summary data for each intervention group and (b) effect estimates and confidence intervals, ideally with a forest plot.	Page 8-10, Table 1
Synthesis of results	21	Present results of each meta-analysis done, including confidence intervals and measures of consistency.	Table 1
Risk of bias across studies	22	Present results of any assessment of risk of bias across studies (see Item 15).	Page 10, Figure 3,4, Table S3
Additional analysis	23	Give results of additional analyses, if done (e.g., sensitivity or subgroup analyses, meta-regression [see Item 16]).	Page 10, Figure 5
**DISCUSSION**			
Summary of evidence	24	Summarize the main findings including the strength of evidence for each main outcome; consider their relevance to key groups (e.g., health care providers, users, and policy makers).	11
Limitations	25	Discuss limitations at study and outcome level (e.g., risk of bias), and at review level (e.g., incomplete retrieval of identified research, reporting bias).	13
Conclusions	26	Provide a general interpretation of the results in the context of other evidence, and implications for future research.	13,14
**FUNDING**			
Funding	27	Describe sources of funding for the systematic review and other support (e.g., supply of data); role of funders for the systematic review.	20

Supplemental table 2 Characteristics of studies included in the meta-analysis

No.	Year	Author	Country	Number	Follow-up time(months)	Cut-off score(H/L)	CD44 antibody	Tumor type	Staining patterns	Clinical stage(I,II/III,IV)	Furhman grade	Reccurence	Microvascular invasion	5-DSS	5-DFS	5-OS
**1**	1998	E de Alava(1)	Spain	58	36-79	1%,23/35	CD44v6	C,P,CH others	membrane	H:12/11; L:25/10	H:9/14 L:30/5	NA	NA	NA	NA	H:11/12: L:27/8
**2**	1999	C. Fischer(2)	Germany	137	52.6(no progression) 18.7(progression)	20%,27/110	CD44s	C	NA	NA	NA	NA	NA	NA	H:6/21; L:67/43	NA
**3**	1999	Michael Z.Gilcrease(3)	United States	43	43	5%,10/22	CD44s	C,P,CH	membrane	H:4/6; L:13/9	H:0/10; L:10/12	H:5/2; L:0/13	NA	NA	NA	NA
						6/26	CD44s			H:0/6; L:17/9	H:2/4; L:8/28	H:2/1; L:3/14	NA	NA	NA	NA
						2/30	CD44v3			H:0/2; L:17/13	H:0/2; L:10/20	H:0/0; L:5/15	NA	NA	NA	NA
						4/28	CD44v6			H:1/3; L:16/12	H:2/2; L:8/20	H:1/1; L:4/14	NA	NA	NA	NA
**4**	1999	Valerie Paradis(4)	France	66	54	1%,32/34	CD44H	C	Membrane, Cytoplasm	NA	H:8/24; L:18/16	NA	NA	NA	NA	H:20/12; L:32/2
						1%,2/64	CD44v6		Membrane, Cytoplasm	NA	NA	NA	NA	NA	NA	NA
**5**	2000	N.LI(5)	Japan	60	48.6	20%,22/38	CD44v10	C,G	Cytoplasm	H:11/11; L:32/6	NA	NA	NA	H:12/10; L:36/2	NA	NA
**6**	2000	Laurent Daniel(6)	France	95	58.1	1%,26/69	CD44	C	Membrane, cytoplasm	NA	H:16/10; L:67/2	NA	NA	NA	H:22/4; L:66/3	NA
**7**	2001	Nathalie Rioux(7) Leclercq	France	73	52	20%,50/23	CD44s	C	Membrane, cytoplasm	NA	NA	NA	NA	H:16/34; L:21/2	NA	NA
**8**	2002	Shengtang Wu(8)	China	67	>60	0%,62/5	CD44v5	C,G,S	Membrane	NA	H:27/35; L:0/5	NA	NA	NA	NA	H:43/19; L:1/4
**9**	2002	A.Bamias(9)	Greece	92	41.5	10%,48/44	CD44s		Membrane, cytoplasm	NA	H:32/16; L:32/12	NA	NA	NA	NA	H:31/17; L:30/14

**10**	2004	Ksenija(10)	Croatia	173	85	1%,70/103	CD44s	C	Membrane, cytoplasm	H:41/28; L:79/25	H:23/47; L:76/27	NA	NA	NA	NA	H:31/39; L:66/37
						11/162	CD44v5	C	Membrane, cytoplasm	NA	NA	NA	NA	NA	NA	NA
						28/145	CD44v6	C	Membrane, cytoplasm	NA	NA	NA	NA	NA	NA	NA
**11**	2004	E. Yildiz(11)	Turkey	42	48	20%,20/22	CD44	C	Membrane, cytoplasm	NA	NA	NA	H:18/2; L:5/17	H:2/18; L:18/4	NA	NA
**12**	2005	Koviljka Matusan(12)	Croatia	38		>1%,22/16	CD44s	P	Membrane, cytoplasm	H:21/1; L:11/5	H:14/8; L:12/4	NA	NA	NA	NA	NA
						12/26	CD44v6	P	Membrane, cytoplasm	NA	NA	NA	NA	NA	NA	NA
**13**	2006	Mahmood Kabiri(13)	Iran	46	31.3	5%,15/31	CD44	C,P,S,P,CH		NA	NA	NA	NA	NA	NA	H:9/6; L:28/3
**14**	2007	Ossam W.Tawfik(14)	United States	62	22	1%,20/42	CD44	C,CH,P	Cytoplasm	NA	NA	NA	NA	NA	NA	H:10/10; L:21/21
**15**	2008	So Dug Lim(15)	Korea	86	46	5%,38/48	CD44s	C,P,CH	Membrane, cytoplasm	H:16/22; L:24/24	H:6/32; L:23/25	NA	NA	NA	H:27/11; L:31/17	NA
**16**	2012	Byung Joo Jeong(16)	Korea	110	60	50%,18/92	CD44	C	Membrane cytoplasm	NA	H:11/7; L:80/12	H:11/7; L:8/84	NA	H:10/8; L:87/5	H:7/11; L:84/8	H:9/9; L:81/11
**17**	2012	Walter Henriques dacosta(17)	Brazil	99	43	50%,42/57	CD44s	C	Membrane, Cytoplasm, nucleus	H:17/23; L:43/16	H:23/19; L:46/11	NA	H:11/29; L:5/54	H:27/13; L:52/7	H:26/16; L:45/12	NA
**18**	2013	Yanhui Zhang(18)	China	110	64.7	1%,30/80	CD44	C,P,CH	membrane	H:8/22; L:49/31	H:6/24; L:55/25	NA	NA	NA	NA	NA
**19**	2014	Jun Qin(19)	China	75	63	25%,35/40	CD44s	C	Membrane, cytoplasm	NA	H:15/20; L:34/6	NA	NA	NA	NA	H:14/9; L:17/3
**20**	2014	Shuji Mikami(20)	Japan	120		11%,27/93	CD44	C	Membrane	NA	NA	NA	NA	NA	H:8/19; L:84/9	H:13/14; L:83/10

Supplemental table 3 Heterogeneity test and publication bias analyses among studies included

	Clinical Stage (I+II vs. III+IV)				Recurrence (positive vs.negative)				MVI (positive vs.negative)				Furhamn Grade (grade I+II vs.grade III+IV)				5-Overall Survival (survive vs. death)				5-Disease Specific Survival (survive vs. death)				5-Disease Free Survival (survive vs. death)			
	Heterogeneity		Publication bias		Heterogeneity		Publication bias		Heterogeneity		Publication bias		Heterogeneity		Publication bias		Heterogeneity		Publication bias		Heterogeneity		Publication bias		Heterogeneity		Publication bias	
	P^a	$I^2(\%)^b$	P^c	P^d	P^a	$I^2(\%)^b$	P^c	P^d	P^a	$I^2(\%)^b$	P^c	P^d	P^a	$I^2(\%)^b$	P^c	P^d	P^a	$I^2(\%)^b$	P^c	P^d	P^a	$I^2(\%)^b$	P^c	P^d	P^a	$I^2(\%)^b$	P^c	P^d
**Over all**	0.002	71.8	0.368	0.337	0.47	0	1	0.821	0.755	0	1		0.001	65.2	0.436	0.194	0.23	24.6	0.602	0.457	0	83.7	0.308	0.125	0	86.3	0.26	0.045
**Geographic area**																												
1.Asia	0.081	67.1	0.317	NA	NA	NA	NA	NA	NA	NA	NA	NA	0.065	58.5	0.308	0.101	0.711	0	0.734	0.567	NA	NA	NA	NA	0	91.3	0.296	0.09
2.Non-Asian	0.002	75.6	0.806	0.582	NA	NA	NA	NA	NA	NA	NA	NA	0.013	63	0.548	0.742	0.112	46.7	1	0.928	0	83.7	0.602	0.291	0.006	80.2	0.296	0.066
**Staining pattern**																												
1.membrane	0.34	7.3	0.602	0.59	NA	NA	NA	NA	NA	NA	NA	NA	0.025	68.1	1	0.673	NA	NA	NA	NA	NA	NA	NA	NA	0	95.8	0.317	NA
2.cytoplasm	NA	NA	NA	NA	NA	NA	NA	NA	NA	NA	NA	NA	NA	NA	NA	NA	NA	NA	NA	NA	NA	NA	NA	NA	NA	NA	NA	NA
3.membrane and cytoplasm	0.006	76.1	1	0.92	NA	NA	NA	NA	NA	NA	NA	NA	0.005	67.5	0.548	0.159	0.209	30.2	0.452	0.174	NA	NA	NA	NA	0.008	79.4	0.602	0.442
**Cutoff of staining**																												
1.<20%	0.001	79.8	0.221	0.12	NA	NA	NA	NA	NA	NA	NA	NA	0.277	21.6	0.707	0.202	0.123	44.9	0.806	0.953	NA	NA	NA	NA	0	94.3	1	NA
2.>=20%	0.195	40.5	1	NA	NA	NA	NA	NA	NA	NA	NA	NA	0	78.4	0.462	0.436	0.589	0	0.734	0.195	NA	NA	NA	NA	0	84.1	0.308	0.006
**Sample size**																												
<58.5	0.006	39.6	0.296	0.095	NA	NA	NA	NA	NA	NA	NA	NA	0.067	58.2	1	0.912	0.44	0	0.806	0.692	0.085	66.3	1	0	NA	NA	NA	NA
≥58.5	0.174	80.5	0.308	0.331	NA	NA	NA	NA	NA	NA	NA	NA	0.001	72.1	0.23	0.039	0.079	55.7	0.308	0.214	0.255	22.8	1	0	NA	NA	NA	NA
**Follow time(month)**																												
<68.5	0.329	12.7	0.734	0.437	NA	NA	NA	NA	NA	NA	NA	NA	0.019	66	0.462	0.418	0.348	0	1	0.826	0.001	90.7	1	0	0	91.7	0.317	0
≥68.5	0.06	71.7	1	0	NA	NA	NA	NA	NA	NA	NA	NA	0.1	48.6	0.462	0.195	0.739	5.4	0.806	0.16	0.086	66	1	0	0.096	63.8	0.602	0.034

a P for heterogeneity within each subgroup.

b Proportion of between-study heterogeneity accounting for total heterogeneity.

c P values of Begg’s test.

d P values of Egger’s test.

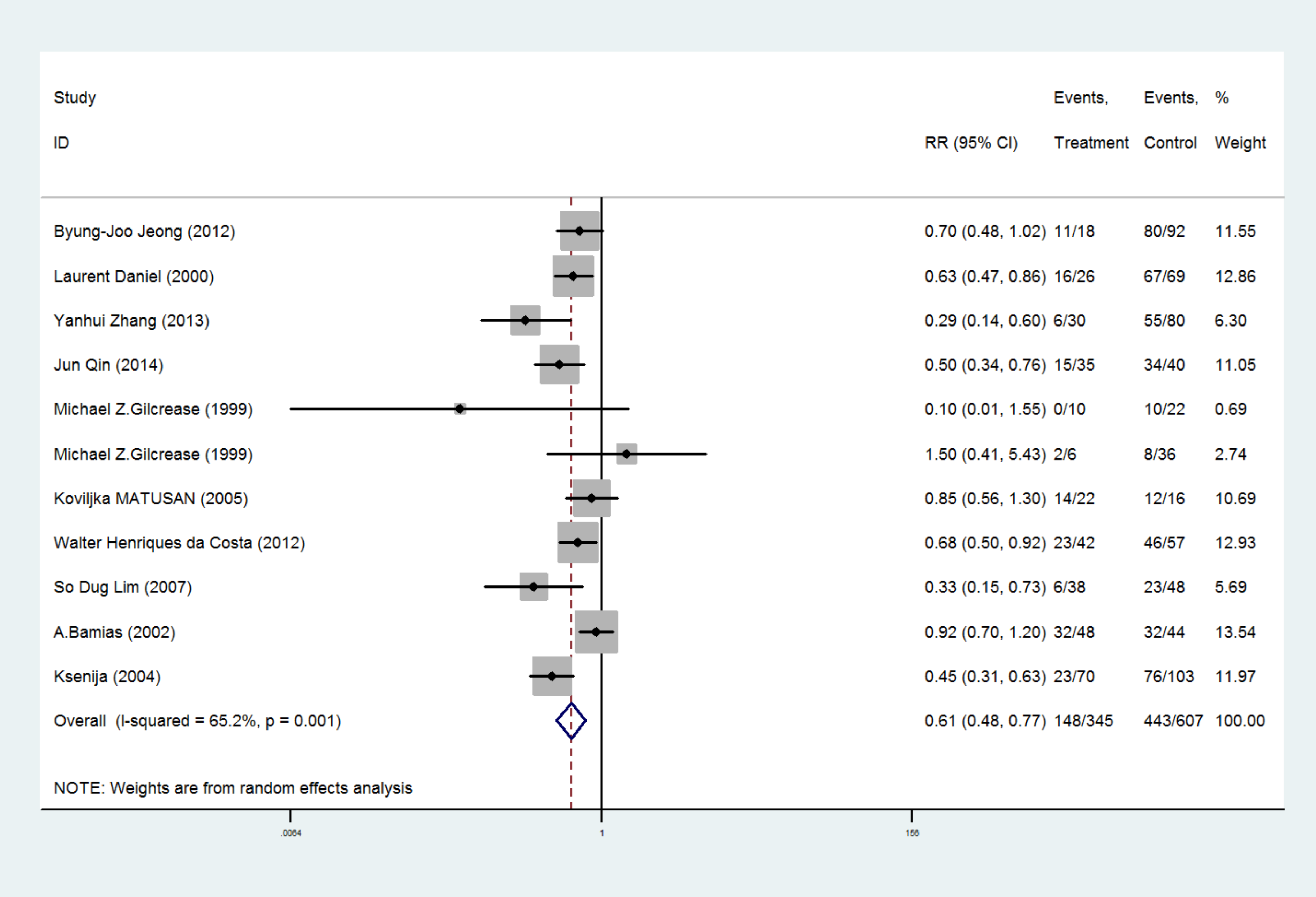

Supplemental figure 1 CD44 expression and Furhman grade

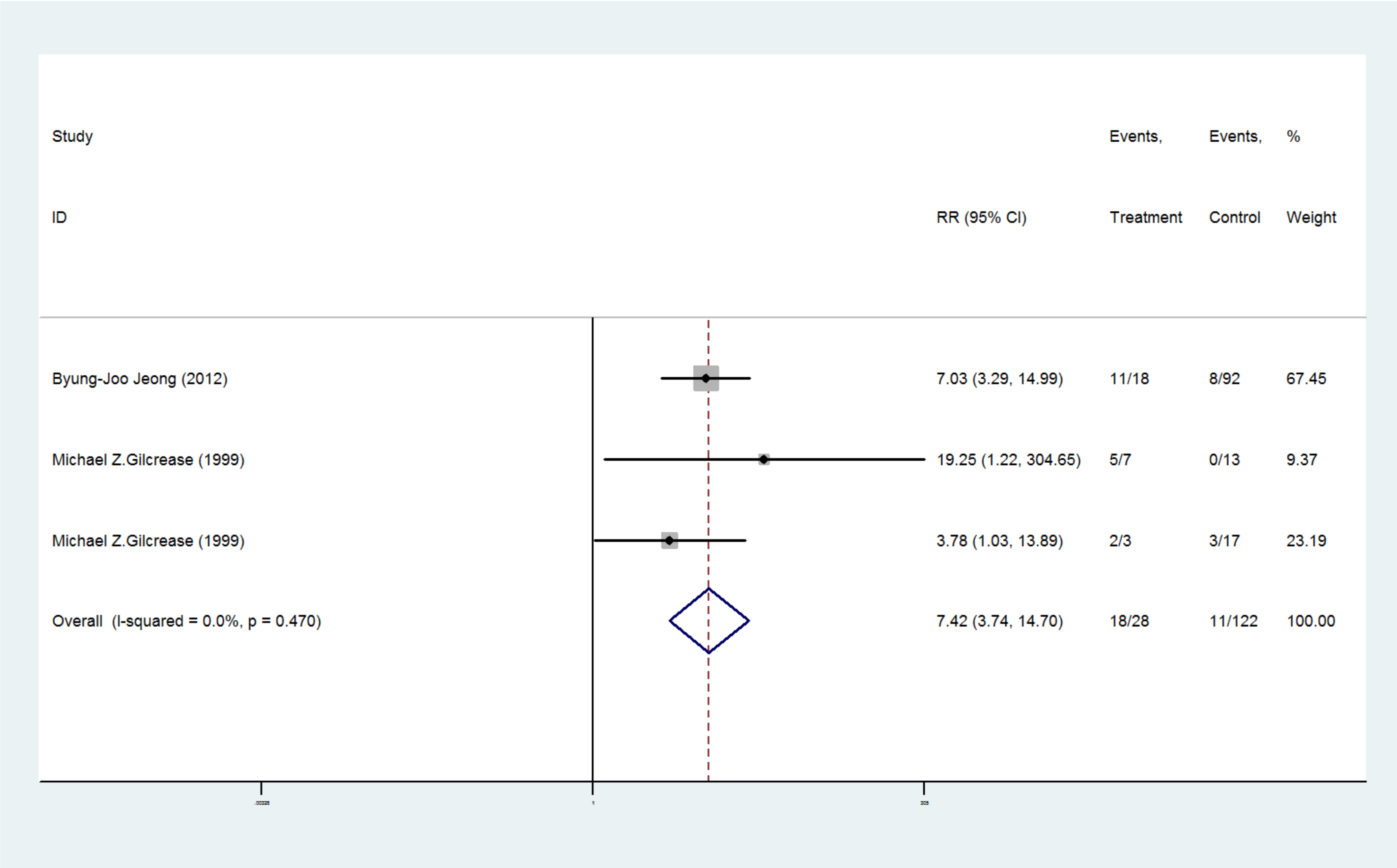

Supplemental figure 2 CD44 expression and tumor recurrence

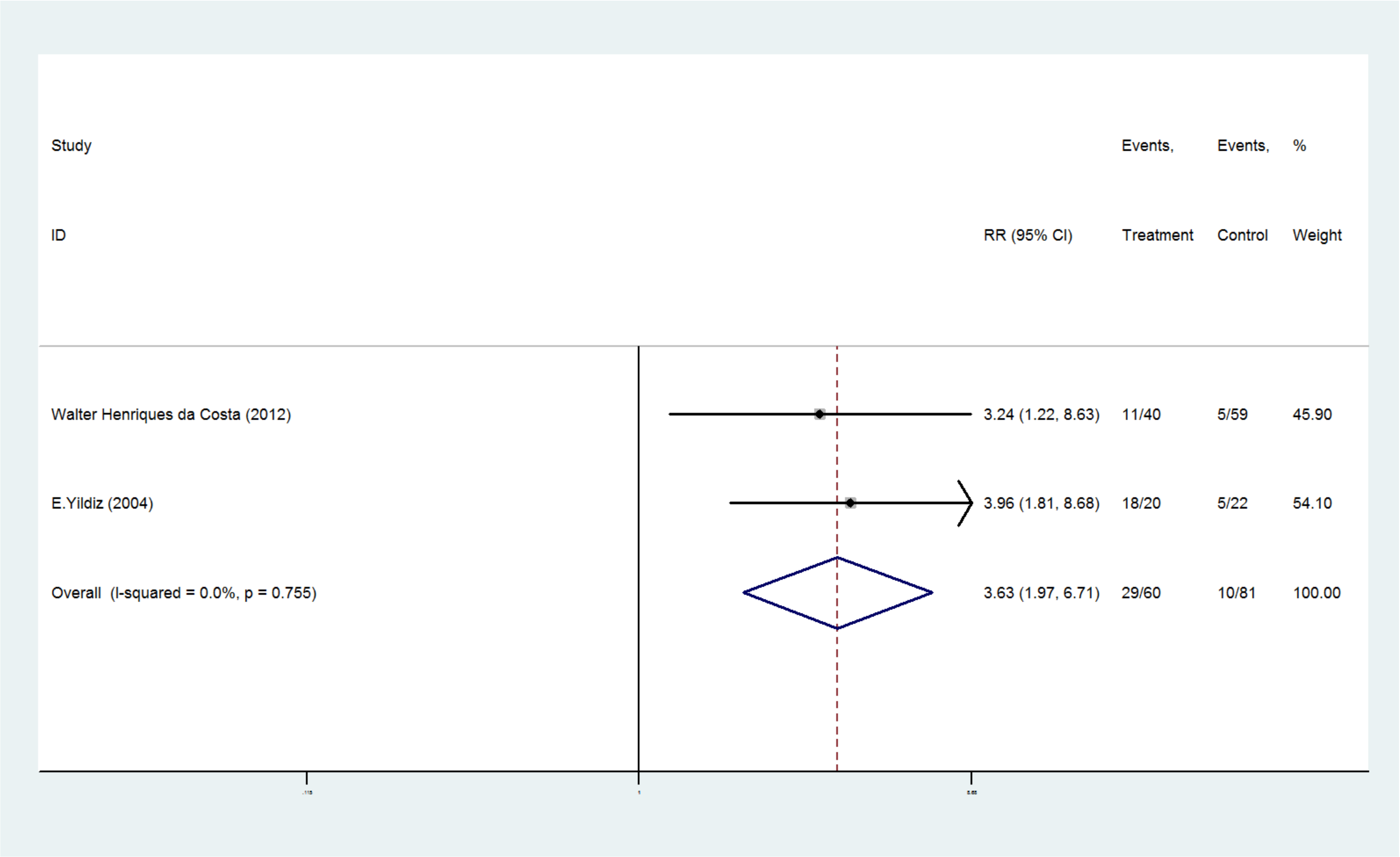

Supplemental figure 3 CD44 expression and tumor MVI

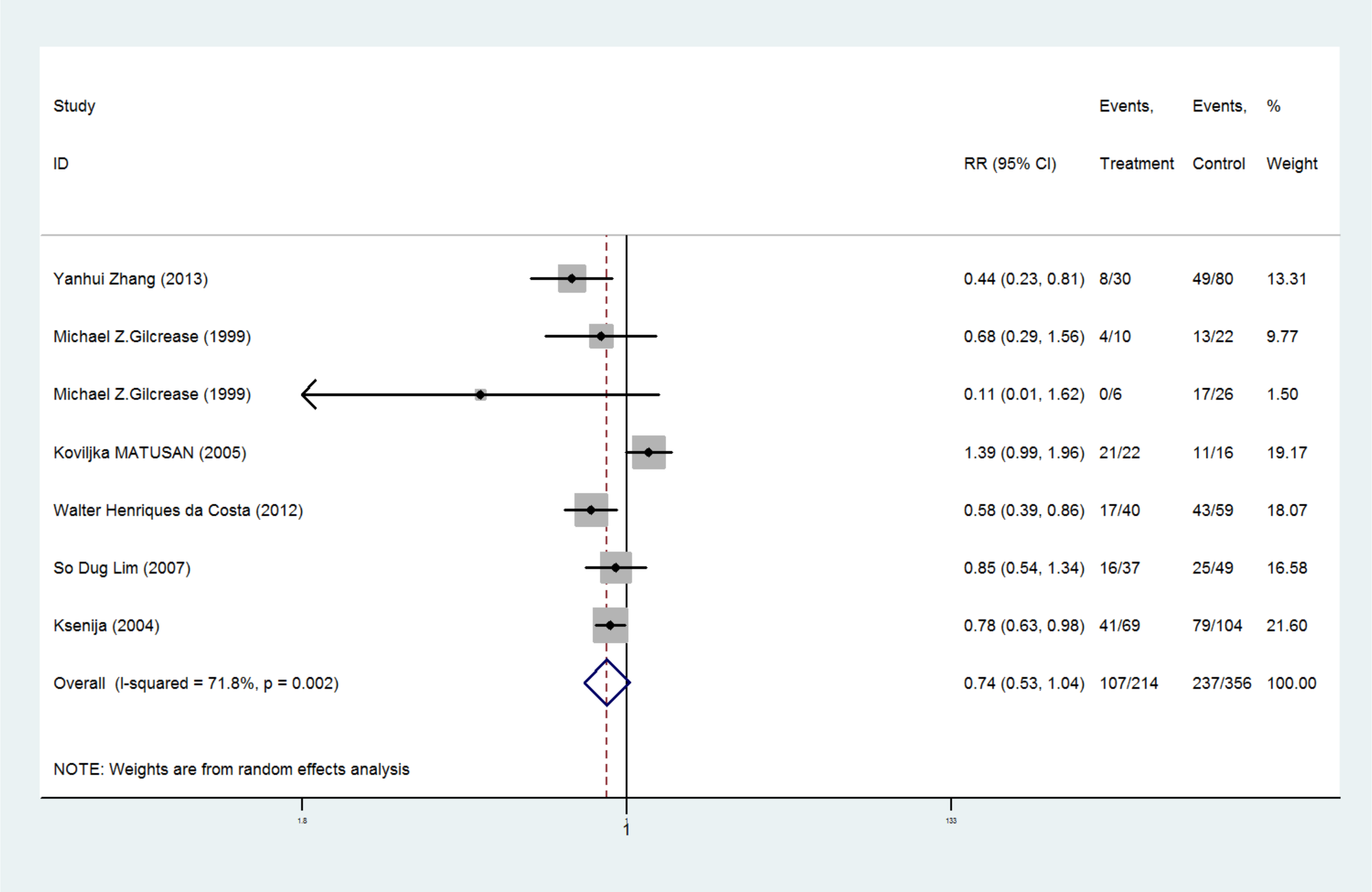

Supplemental figure 4 CD44 expression and tumor clinical stage

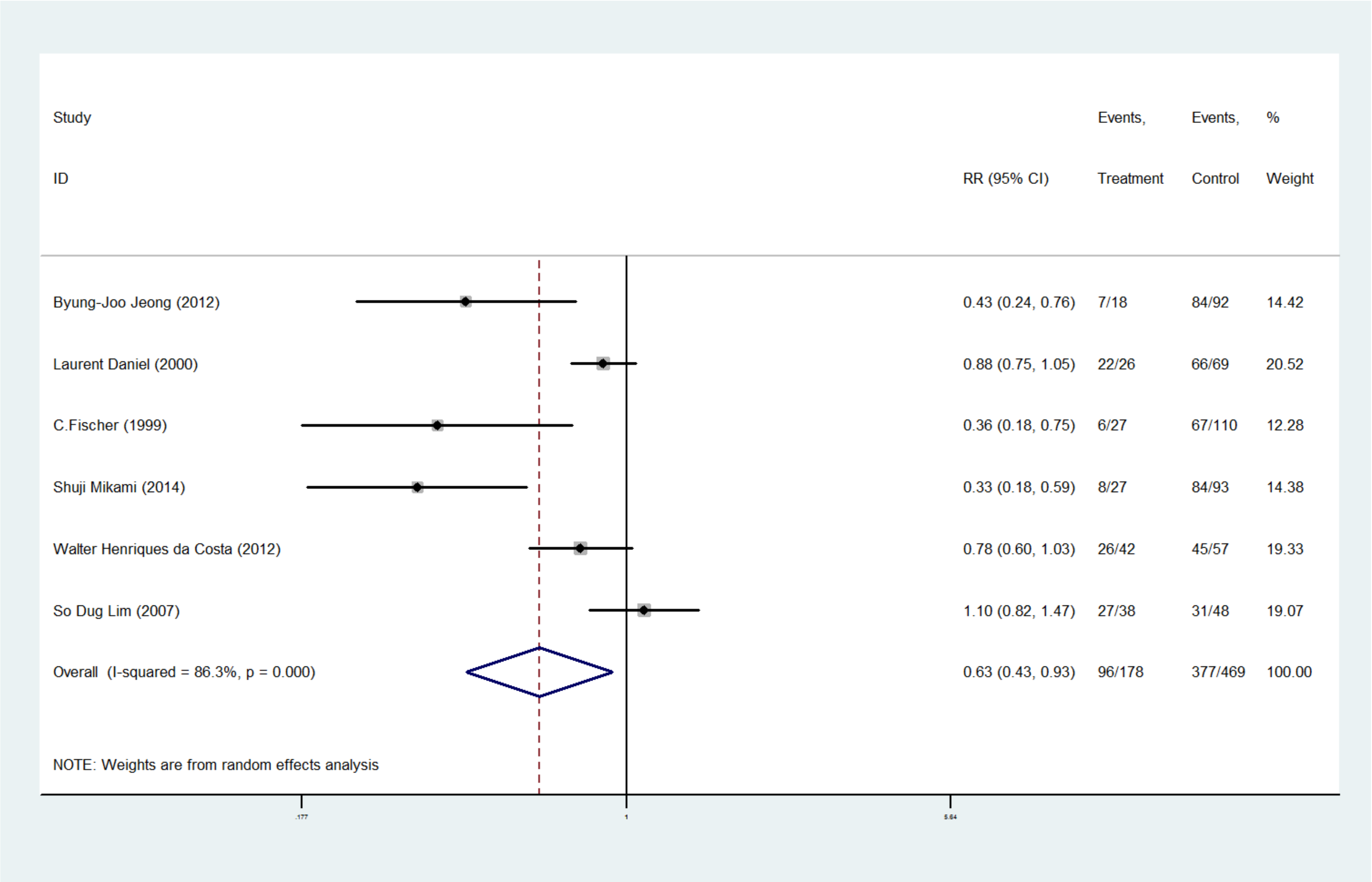

Supplemental figure 5 CD44 expression and 5-year DFS

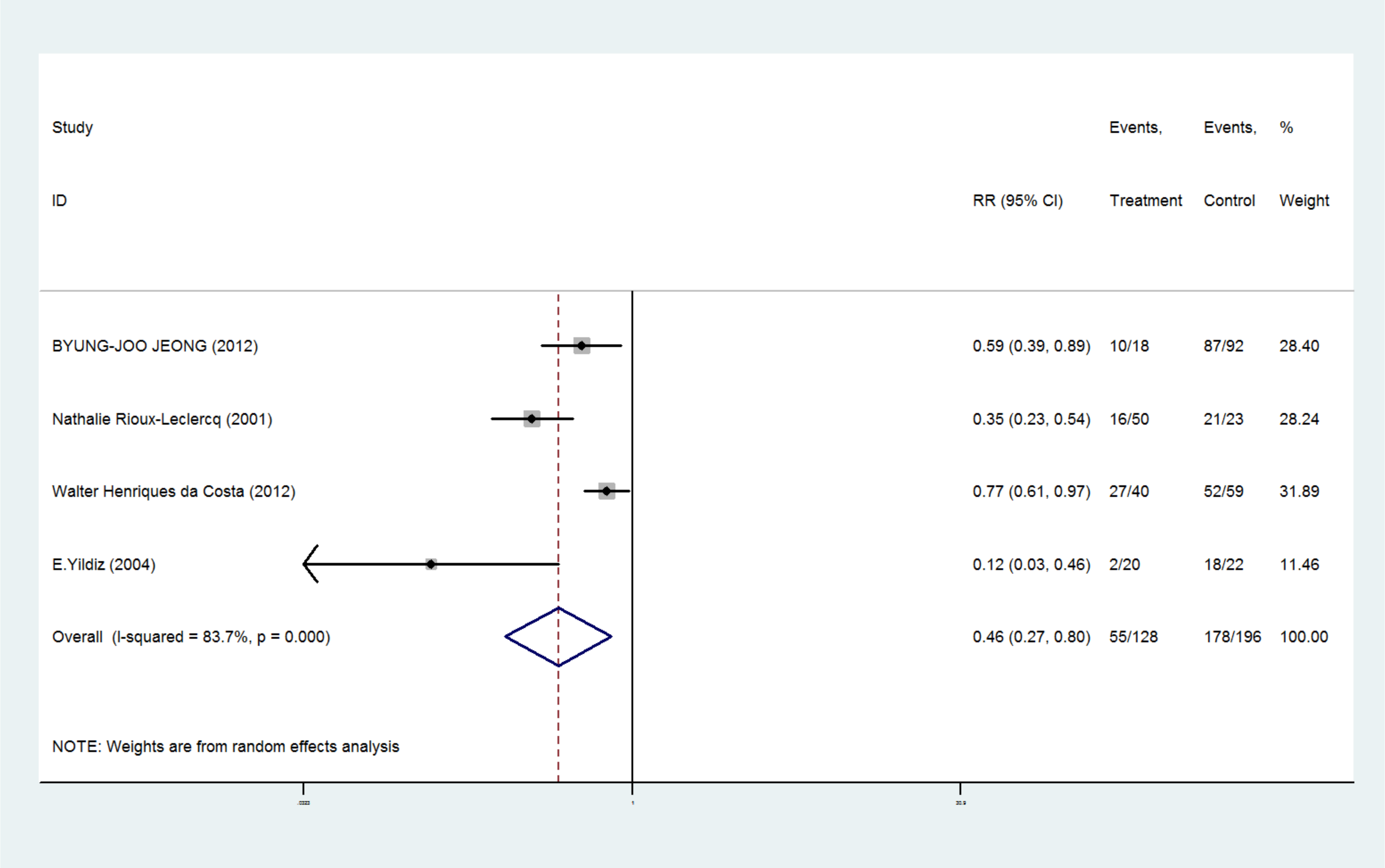

Supplemental figure 6 CD44 expression and 5-year DSS

References

1 de Alava, E., Panizo, A., Sola, I., Rodriguez-Rubio, F. I. & Pardo-Mindan, F. J. CD44v6 expression is related to progression in renal epithelial tumours. *Histopathology* **33**, 39-45 (1998).

2 Fischer, C. *et al.* CD44s, E-cadherin and PCNA as markers for progression in renal cell carcinoma. *Anticancer research* **19**, 1513-1517 (1999).

3 Gilcrease, M. Z. *et al.* Correlation of CD44S expression in renal clear cell carcinomas with subsequent tumor progression or recurrence. *Cancer* **86**, 2320-2326 (1999).

4 Paradis, V. *et al.* CD44 is an independent prognostic factor in conventional renal cell carcinomas. *The Journal of urology* **161**, 1984-1987 (1999).

5 Li, N. *et al.* Analysis of CD44 isoform v10 expression and its prognostic value in renal cell carcinoma. *BJU international* **85**, 514-518 (2000).

6 Daniel, L. *et al.* CD44s and CD44v6 expression in localized T1-T2 conventional renal cell carcinomas. *The Journal of pathology* **193**, 345-349, doi:10.1002/1096-9896(2000)9999:9999<::aid-path817>3.0.co;2-h (2001).

7 Rioux-Leclercq, N. *et al.* Clinical significance of cell proliferation, microvessel density, and CD44 adhesion molecule expression in renal cell carcinoma. *Human pathology* **32**, 1209-1215 (2001).

8 Wu, S. T. *et al.* Correlation of CD44v5 expression with invasiveness and prognosis in renal cell carcinoma. *Journal of the Formosan Medical Association = Taiwan yi zhi* **102**, 229-233 (2003).

9 Bamias, A. *et al.* Prognostic significance of CA 125, CD44, and epithelial membrane antigen in renal cell carcinoma. *Urology* **62**, 368-373 (2003).

10 Lucin, K., Matusan, K., Dordevic, G. & Stipic, D. Prognostic significance of CD44 molecule in renal cell carcinoma. *Croatian medical journal* **45**, 703-708 (2004).

11 Yildiz, E. *et al.* Prognostic value of the expression of Ki-67, CD44 and vascular endothelial growth factor, and microvessel invasion, in renal cell carcinoma. *BJU international* **93**, 1087-1093, doi:10.1111/j.1464-410X.2004.04786.x (2004).

12 Matusan, K., Dordevic, G., Mozetic, V. & Lucin, K. Expression of osteopontin and CD44 molecule in papillary renal cell tumors. *Pathology oncology research : POR* **11**, 108-113, doi:Paor.2005.11.2.0108 (2005).

13 Mahnood Kabiri, M. M. S., Diana Taheri, Ali Chehrei. Prognostic value of CD44 in renal cell carcinoma. *J Res Med Sci* **11**, 5 (2006).

14 Tawfik, O. W. *et al.* Prognostic significance of CD44, platelet-derived growth factor receptor alpha, and cyclooxygenase 2 expression in renal cell carcinoma. *Archives of pathology & laboratory medicine* **131**, 261-267, doi:10.1043/1543-2165(2007)131[261:psocpg]2.0.co;2 (2007).

15 Lim, S. D., Young, A. N., Paner, G. P. & Amin, M. B. Prognostic role of CD44 cell adhesion molecule expression in primary and metastatic renal cell carcinoma: a clinicopathologic study of 125 cases. *Virchows Archiv : an international journal of pathology* **452**, 49-55, doi:10.1007/s00428-007-0530-4 (2008).

16 Jeong, B. J. *et al.* CD44 is associated with tumor recurrence and is an independent poor prognostic factor for patients with localized clear cell renal cell carcinoma after nephrectomy. *Experimental and therapeutic medicine* **3**, 811-817, doi:10.3892/etm.2012.505 (2012).

17 Costa, W. H., Rocha, R. M., Cunha, I. W., Guimaraes, G. C. & Zequi Sde, C. Immunohistochemical expression of CD44s in renal cell carcinoma lacks independent prognostic significance. *International braz j urol : official journal of the Brazilian Society of Urology* **38**, 456-465 (2012).

18 Zhang, Y. *et al.* Clinical significances and prognostic value of cancer stem-like cells markers and vasculogenic mimicry in renal cell carcinoma. *Journal of surgical oncology* **108**, 414-419, doi:10.1002/jso.23402 (2013).

19 Qin, J. *et al.* Concurrent CD44s and STAT3 expression in human clear cell renal cellular carcinoma and its impact on survival. *International journal of clinical and experimental pathology* **7**, 3235-3244 (2014).

20 Mikami, S. *et al.* Expression of TNF-alpha and CD44 is implicated in poor prognosis, cancer cell invasion, metastasis and resistance to the sunitinib treatment in clear cell renal cell carcinomas. *International journal of cancer. Journal international du cancer*, doi:10.1002/ijc.29137 (2014).
